# Age-associated changes in DNA methylation across multiple tissues in an inbred mouse model

**DOI:** 10.1016/j.mad.2016.02.001

**Published:** 2016-03

**Authors:** Helen Spiers, Eilis Hannon, Sara Wells, Brenda Williams, Cathy Fernandes, Jonathan Mill

**Affiliations:** a Social, Genetic and Developmental Psychiatry Centre, Institute of Psychiatry, Psychology and Neuroscience, King’s College London, London SE5 8AF, UK; b University of Exeter Medical School, University of Exeter, Exeter EX2 5DW, UK; c Mary Lyon Centre, Harwell, Didcot, Oxfordshire OX11 ORD, UK; d Department of Basic and Clinical Neuroscience, Institute of Psychiatry, Psychology and Neuroscience, King’s College London, London SE5 8AF, UK

**Keywords:** Aging, Epigenetics, DNA methylation, Inbred mouse, Cross-tissue

## Abstract

Epigenetic disruption has been implicated in many diseases of aging, and age-associated DNA methylation changes at specific genomic loci in humans are strongly correlated with chronological age. The aim of this study was to explore the specificity of selected age-associated differentially methylated positions (aDMPs) identified in human epidemiological studies by quantifying DNA methylation across multiple tissues in homologous regions of the murine genome. We selected four high-confidence aDMPs (located in the vicinity of the *ELOVL2*, *GLRA1*, *MYOD1* and *PDE4C* genes) and quantified DNA methylation across these regions in four tissues (blood, lung, cerebellum and hippocampus) from male and female C57BL/6J mice, ranging in age from fetal (embryonic day 17) to 630 days. We observed tissue-specific age-associated changes in DNA methylation that was directionally consistent with those observed in humans. These findings lend further support to the notion that changes in DNA methylation are associated with chronological age and suggest that these processes are often conserved across tissues and between mammalian species. Our data highlight the relevance of utilizing model systems, in which environmental and genetic influences can be carefully controlled, for the further study of these phenomena.

## Highlights

• DNA methylation at specific loci is correlated with chronological age in humans.
• Age-associated DNA methylation changes are also seen at selected loci in mouse.
• Link between DNA methylation and age may be conserved across tissues and species.
• Supports the relevance of murine models for further study of the aging epigenome.

Aging, the progressive decline in physiological and psychological functioning that occurs across the lifespan, involves a complex suite of molecular changes (Lopez-Otin et al., 2013) including perturbations to the epigenetic processes regulating gene transcription (Jones et al., 2015). A growing literature, for example, describes robust age-associated DNA methylation changes at specific genomic loci in humans, representing a so-called “epigenetic clock” that is strongly correlated with chronological age (Horvath, 2013). Notably, some changes in DNA methylation associated with age are cell-type specific (Day et al., 2013) while others occur across multiple tissues (Horvath, 2013). Epigenetic changes have been implicated in many diseases of aging including cancer (Bergman and Cedar, 2013) and dementia (Lunnon et al., 2014), and it has been hypothesized that accelerated aging of the “epigenetic clock” is associated with mortality-linked markers of physical and mental fitness (Marioni et al., 2015). Our knowledge about the origins and function of age-associated epigenetic variation remains limited, in part because of the difficulties inherent in studying such dynamic and tissue-specific processes in human cohorts (Heijmans and Mill, 2012). The aim of this study was to explore the specificity of selected age-associated differentially methylated positions (aDMPs) identified in human epidemiological studies by quantifying DNA methylation across multiple tissues in homologous regions of the murine genome.

We aged a colony of inbred C57BL/6J mice and sequentially collected four tissues (whole blood, lung, cerebellum and hippocampus (Table C.1 of Supplementary material) from fetal (embryonic (E) 17, -4 days old) to elderly (630 days old) individuals (Fig. B.1 of Supplementary material). Targeted assays were designed to quantify DNA methylation across regions of the murine genome homologous to four robustly-associated human aDMPs in the vicinity of the genes *ELOVL*2, *GLRA1*, *MYOD*1 and *PDE4C* (Table C.2, Figs. B.2–B.5 of Supplementary material) that have been previously associated with chronological age (Bell et al., 2012, Bocklandt et al., 2011, Florath et al., 2014, Garagnani et al., 2012, Hannum et al., 2013, Hernandez et al., 2011, Johansson et al., 2013, Koch and Wagner, 2011, Rakyan et al., 2010, Teschendorff et al., 2010, Weidner et al., 2014). Briefly, genomic DNA was treated with sodium bisulfite, and DNA methylation was quantified across multiple CpG sites using the Sequenom EpiTYPER system following bisulfite-PCR amplification. A full description of experimental methods is given in Appendix A.

**Fig. 1:**
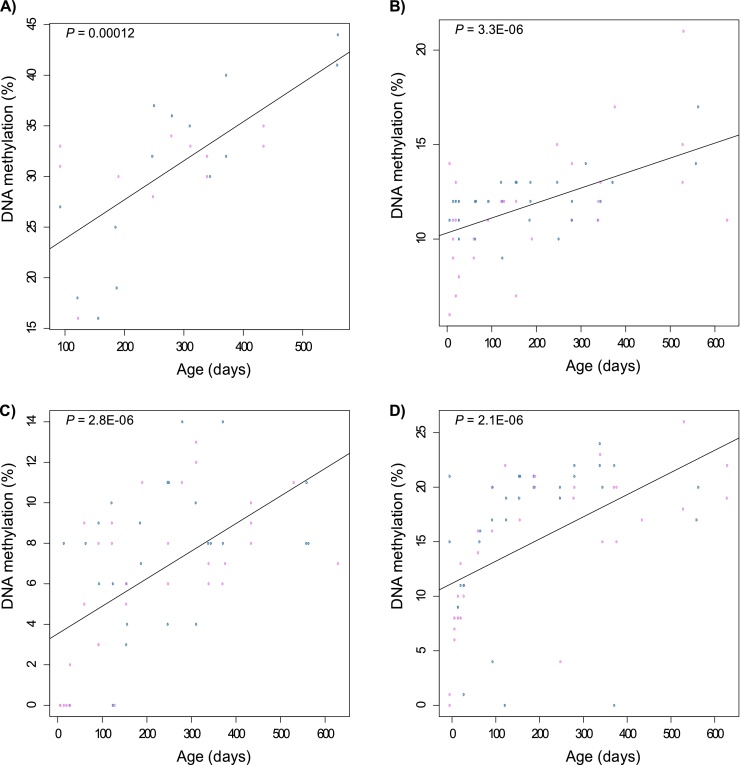
DNA methylation across regions homologous to human aDMP is associated with chronological age in mouse. For each of the amplicons the most significantly age associated CpG site across the four tissues assessed is shown. (A) ELOVL2 – CpG sites 2/3 – blood (*P* = 1.15E − 04). (B) GLRA1 – CpG sites 13/14 – blood (*P* = 3.31E-06). (C) MYOD1 – CpG site 1 – blood (*P* = 2.82E − 06). D) PDE4C – CpG sites 21/21 – cerebellum (*P* = 2.14E − 06). Blue dots depict male samples, pink dots depict female samples (see also Tables C.7–C.10 of Supplementary material). (For interpretation of the references to colour in this figure legend, the reader is referred to the web version of this article.)

Average and CpG site-specific DNA methylation across the four amplicons in each tissue is shown in Tables C.3–C.6 of Supplementary material. Age-associated changes in DNA methylation were identified using a linear model for each of the four tissues (Table 1 and Tables C.7–C.10 of Supplementary material). Our initial analyses focused on whole blood, the predominant tissue used for epigenetic aging studies in human populations. Average DNA methylation across two amplicons (ELOVL2, *P* = 0.01; GLRA1, *P* = 3.86E − 05) was found to be significantly associated with age in the same direction as reported in human data, with individual CpG units within each amplicon being strongly associated with age (Fig. 1 and Tables C.7–C.8 of Supplementary material). Although amplicon-average DNA methylation across the other two regions was not significantly associated with age in whole blood (MYOD1, *P* = 0.09; PDE4C, *P* = 0.83), multiple CpG units within both amplicons were significantly correlated with age in the direction predicted from human studies (Tables C.9–C.10 of Supplementary material). Together, these data provide evidence that human blood aDMPs are also associated with chronological age in mouse.

We next examined changes in DNA methylation with age at these four loci in three additional tissues dissected from the same individual animals. Amplicon average DNA methylation was associated with age in lung across both the ELOVL2 and GLRA1 amplicons, reflecting the patterns seen in whole blood (ELOVL2, *P* = 0.02; GLRA1, *P* = 0.01), although not in cerebellum or hippocampus (Tables C.7–C.8 of Supplementary material). In contrast, cerebellum-specific associations with age were observed for amplicon-average DNA methylation across the two other amplicons (MYOD1, *P* = 0.02; PDE4C, *P* = 5.74E − 06) (Tables C.9–C.10 of Supplementary material).

These findings lend further support to the notion that changes in DNA methylation are associated with chronological age and suggest that these processes may often be conserved across tissues and between species (Polanowski et al., 2014). Characterization of the molecular mechanisms underpinning normative aging processes has the potential to facilitate the development of novel therapeutic interventions targeting diseases of aging, potentially increasing the health-span of our aging population. Our data highlight the relevance of utilizing model systems, in which environmental and genetic influences can be carefully controlled, for the further study of these phenomena.

## Author contributions

JM and HS conceived the project. CF, SW and BP performed mouse work. HS performed DNA methylation quantification and analysis with advice from EH. HS and JM wrote manuscript. All authors approved the manuscript before submission.

## Conflict of interest

We certify that there is no conflict of interest with any financial organization regarding the material discussed in the manuscript.

## Figures and Tables

**Table 1:** Tissue-specific age-associated changes in DNA methylation were observed candidate regions in an inbred strain of mouse. B = blood; L = lung; C = cerebellum; H = hippocampus.

Amplicon	ELOVL2				GLRA1				MYOD1				PDE4C			
Human aDMP (corresponding illumina 450 K array probe) Human Feb. 2009 (GRCh37/hg19)	cg16867657 Chr6: 11044877				cg00059225 Chr5: 151304357				cg18555440 Chr11: 17741687				cg17861230 Chr19: 18343901			
Homologous mouse target region Mouse July 2007 (NCBI37/mm9)	Chr13: 41316038–41316469				Chr11: 55421383–55421670				Chr7: 53632317–53632673				Chr8: 73253999–73254240			
CpG units passing QC (*n*)	8				10				19				11			
Tissue	B	L	C	H	B	L	C	H	B	L	C	H	B	L	C	H
Amplicon average *P*-value	0.01^*^	0.02^*^	0.18	0.53	3.86E − 05^*^	0.01^*^	0.16	0.59	0.09	0.45	0.02^*^	0.97	0.83	0.89	5.74E − 06^*^	0.59
Age-associated CpG units (*n P* < 0.05) in same direction as human	3	3	1	0	6	4	0	1	5	0	1	0	1	0	6	0
