# Supplementary material for: Age-associated changes in DNA methylation across multiple tissues in an inbred mouse model
Source: Mech Ageing Dev. 2016 Mar;154:20–3. doi: 10.1016/j.mad.2016.02.001 (PMC4798846; doi:10.1016/j.mad.2016.02.001)
Supplement: Supplementary file 1 [file mmc1.docx]

**Appendix A. Experimental procedures**

**Aging mouse samples**

The study used an aging sample of inbred C57BL/6J mice (n = 117: E17 - 630 days old) (**Figure B.1**). Mice were bred and maintained in the Biological Services Unit at the Institute of Psychiatry, Psychology and Neuroscience, King’s College London or the Mary Lyon Centre, Harwell. Mice were housed in standard conditions with ad libitum access to water and food. The housing and test rooms were maintained at constant room temperature (21°C) and humidity (45%) and kept under a regular 12:12 hour light/dark schedule. All procedures were performed in compliance with the local ethical review panel of King’s College London and the Mary Lyon Centre, Harwell and the U.K. Home Office Animals Scientific Procedures Act 1986. Prior to sacrifice, whole blood (~100 µl) was collected in a capillary tube by tail vessel sampling. Postnatal samples were sacrificed by neck dislocation and decapitation, whereas fetal samples were sacrificed by decapitation and dissected in Hank’s balanced solution (Sigma H9394). Dissected tissues were snap frozen in dry ice before being stored at -80°C. Genomic DNA was extracted from whole blood using the QIAamp DNA Micro Kit (Catalogue number 56304, QIAGEN, Germany). A standard phenol-chloroform extraction was used to extract genomic DNA from the lung, cerebellum and hippocampus. Sample quality was assessed using spectrophotometry and agarose gel electrophoresis.

**DNA methylation analysis**

Regions homologous to robustly-associated human aDMPs were targeted in the mouse genome (**Table C.2**). Bisulfite-PCR amplicons were designed using Sequenom EpiDesigner software (www.epidesigner.com). The chained alignment region (most similar to the human aDMP) was included in each amplicon (**Table C.2** and **Figures B.2 - B.5**). Genomic DNA samples (500 ng) were sodium bisulfite treated using the Zymo EZ DNA Methylation-Lightning Kit™ (Zymo Research, CA, USA) following the manufacturer’s standard protocol. DNA methylation was then quantified using the Sequenom EpiTYPER platform. Bisulfite-PCR was performed in duplicate and products pooled prior to subsequent analyses. Positive controls, including both artificially methylated and artificially unmethylated samples were included in all experimental procedures to ensure unambiguous PCR amplification of bisulfite-treated samples. Data generated from the EpiTYPER software were filtered using stringent quality control parameters, and CpG units with low call rates and/or individuals with a high number of missing CpG units were removed.

**Statistical Analysis**

The R statistical computing environment (R 3.0.2) was used to implement analyses to assess age-associated DNA methylation changes across each of the four target regions in each tissue. To assess the relationship between DNA methylation and age for each amplicon, a linear model including the covariates of age and sex was applied to each CpG unit and the amplicon average. This model was applied to each tissue separately, allowing identification of tissue-specific age-associated DNA methylation.
