# Supplementary material for: Age-associated changes in DNA methylation across multiple tissues in an inbred mouse model
Source: Mech Ageing Dev. 2016 Mar;154:20–3. doi: 10.1016/j.mad.2016.02.001 (PMC4798846; doi:10.1016/j.mad.2016.02.001)
Supplement: Supplementary file 2 [file mmc2.docx]

**Appendix B - Supplementary Figures**

**Fig. B.1 - Age distribution of the C57BL/6J mice used in this study.** See also **Table C.1**.

Fig. B.2 - ELOVL2 amplicon

UCSC genome browser viewer shows the sequence from Mouse (Chr13: 41315347 - 41316502) targeted using the Sequenom EpiDesigner software, the chained alignment of the Illumina 450K array probe sequence (Chr13: 41316074 - 41316131), and the Sequenom EpiTYPER amplicon targeted by bisulfite-PCR (Chr13: 41316038 - 41316469) (Mouse July 2007 (NCBI37/mm9).

**Fig. B.3 - GLRA1 amplicon**

UCSC genome browser viewer shows the sequence from Mouse (Chr11: 55421016 - 55421973) targeted using the Sequenom EpiDesigner software, the chained alignment of the Illumina 450K array probe sequence (Chr11: 55421477 - 55421518), and the Sequenom EpiTYPER amplicon targeted by bisulfite-PCR (Chr11: 55421383 - 55421670) (Mouse July 2007 (NCBI37/mm9)).

**Fig. B.4 - MYOD1 amplicon**

UCSC genome browser viewer shows the sequence from Mouse (Chr7: 53631909 - 53632854) targeted using the Sequenom EpiDesigner software, the chained alignment of the Illumina 450K array probe sequence (Chr7: 53632400 - 53632449), and the Sequenom EpiTYPER amplicon targeted by bisulfite-PCR (Chr7: 53632317 - 53632673) (Mouse July 2007 (NCBI37/mm9)).

**Fig. B.5 - PDE4C amplicon**

UCSC genome browser viewer shows the sequence from Mouse (Chr8: 73253745 - 73254501) targeted using the Sequenom EpiDesigner software, the chained alignment of the Illumina 450K array probe sequence (Chr8: 73254068 - 73254117) and the Sequenom EpiTYPER amplicon targeted by bisulfite-PCR (Chr8: 73253999 - 73254240) (Mouse July 2007 (NCBI37/mm9)).
