# Supplementary material for: Age-associated changes in DNA methylation across multiple tissues in an inbred mouse model
Source: Mech Ageing Dev. 2016 Mar;154:20–3. doi: 10.1016/j.mad.2016.02.001 (PMC4798846; doi:10.1016/j.mad.2016.02.001)
Supplement: Supplementary file 3 [file mmc3.docx]

**Appendix C. Supplementary Tables.**

**Table C.1 - Samples profiled in this study for each of the four amplicons**

| **Amplicon** | **ELOVL2** | | | **GLRA1** | | | **MYOD1** | | | **PDE4C** | | |
| --- | --- | --- | --- | --- | --- | --- | --- | --- | --- | --- | --- | --- |
| **Samples** | **Total** | **Females** | **Males** | **Total** | **Females** | **Males** | **Total** | **Females** | **Males** | **Total** | **Females** | **Males** |
| **Total** n  (age range in days) | 202  (-4, 630) | 105  (-4, 630) | 97  (-4, 564) | 233  (-4, 630) | 124  (-4, 630) | 109  (-4, 564) | 225  (-4, 630) | 120  (-4, 630) | 105  (-4, 564) | 304  (-4, 630) | 153  (-4, 630) | 151  (-4, 564) |
| **Blood** n  (age range in days) | 25  (93, 560) | 11  (93, 435) | 14  (93, 560) | 59  (7, 629) | 28  (7, 629) | 31  (7, 564) | 60  (7, 630) | 32  (7, 630) | 28  (7, 564) | 69  (7, 629) | 33  (7, 629) | 36  (7, 564) |
| **Lung** n  (age range in days) | 41  (7, 629) | 22  (7, 629) | 19  (15, 372) | 64  (7, 630) | 34  (7, 630) | 30  (7, 564) | 44  (7, 630) | 25  (7, 630) | 19  (7, 560) | 88  (7, 630) | 45  (7, 630) | 43  (7, 560) |
| **Cerebellum** n  (age range in days) | 55  (-4, 630) | 28  (7, 630) | 27  (-4, 564) | 55  (7, 630) | 29  (7, 630) | 26  (7, 564) | 56  (-4, 630) | 27  (-4, 630) | 29  (-4, 564) | 68  (-4, 630) | 34  (-4, 630) | 34  (-4, 564) |
| **Hippocampus** n  (age range in days) | 81  (-4, 630) | 44  (-4, 630) | 37  (-4, 564) | 55  (-4, 629) | 33  (-4, 629) | 22  (-4, 560) | 65  (-4, 629) | 36  (-4, 629) | 29  (-4, 560) | 79  (-4, 629) | 41  (-4, 629) | 38  (-4, 564) |

**Table C.2 - Sequenom PCR primers and target sequence.** Left primer tag = aggaagagag, right primer tag = cagtaatacgactcactatagggagaaggct; Tm = melting temperature. CpG sites are highlighted in red and numbered sequentially. Sequences homologous to Illumina 450K array probes targeting human aDMPs are highlighted in yellow.

|  | **Forward primer** | **Tm** | **Target sequence in murine genome** | **Product**  **size (bp)** |
| --- | --- | --- | --- | --- |
|  | **Reverse primer** |  |  |  |
| **ELOVL2** | GGTTGGGATTTGTAAGTATAGTTGG | 59 | GGCTGGGATTTGCAAGCACAGCTGGCG^1^CCTTTTGCAGGAGTCTGGGCG^2^CG^3^CACG^4^TCTATCACAGCATCAGGCTCCTGGAGAAGCCACCAGCAGCTCCTCCG^5^CTACTCCTGGGGAGTGGCTGGGCG^6^AGCG^7^CG^8^CCG^9^GATGATTTAAATTGGGCACACG^10^GGTTTGAGGGGATGACCAAGGGCTGGTCTGGTCTTGGGTTGGTGCACCTGTATAATCCCAGCACTTGAGAGCAGAGCCAGGAGGAATAGGAAAGAGTTCAAGATCAGCCTTGGCTTCTTAGGGAGTTCTGCACCAACCTGAGCTTCG^11^TGAGACCCTGCTTCAAATAAGCAAACAAATAAGCAAACG^12^ATAACCATTCATTCTGGGTGCCACG^13^GGGAGCAGATGAGCACCAATAGTCTGTCTAAACTGACTTTTTCAGAGTTTGCTTCCAGAGGGGCT | 432 |
|  | AACCCCTCTAAAAACAAACTCTAAAA | 59 |  |  |
| **GLRA1** | GGGGGAGAATTGTAGTTAGGTAGTT | 59 | GGGGGAGAATTGCAGCCAGGCAGCTAAGGTACCG^1^CG^2^GACAGCG^3^GCAGTCG^4^TGCAGGTTCAGCACCACG^5^GAGAGCG^6^TCCAGCCCG^7^GCTGCG^8^GGCG^9^GGAAGAGCG^10^CG^11^GCTGAAGCTCTACTCG^12^CTGGAGGCTCCCTGCG^13^GCG^14^CTGGGAGGCACG^15^TTTGGGGGTGGGTATATTTTCTTCTTTTCTTTCTTTCTTTACTCTTCTTCTTCTTTATTTTTACCCTTATTTTCCTGTCG^16^AGATGGCCAGCTCCATGAAAGGAAGAAATCCG^1^7TGGGGATCAGAGCCCTGGCCTCAG | 288 |
|  | CTAAAACCAAAACTCTAATCCCCA | 60 |  |  |
| **MYOD1** | GTTGTTTGTTGTGGGTTTGTAAG | 59 | GCTGCTTGCTGTGGGCCTGCAAGGCG^1^TGCAAGCG^2^CAAGACCACCAACG^3^CTGATCG^4^CCG^5^CAAGGCCG^6^CCACCATGCG^7^CG^8^AGCG^9^CCG^10^CCG^11^CCTGAGCAAAGTGAATGAGGCCTTCG^12^AGACG^13^CTCAAGCG^14^CTGCACG^15^TCCAGCAACCCG^16^AACCAGCG^17^GCTACCCAAGGTGGAGATCCTGCG^18^CAACG^19^CCATCCG^20^CTACATCG^21^AAGGTCTGCAGGCTCTGCTGCG^22^CG^23^ACCAGGACG^24^CCG^25^CG^26^CCCCCTGGCG^27^CCG^28^CTGCCTTCTACG^29^CACCTGGACCG^30^CTGCCCCCAGGCCG^31^TGGCAGCG^32^AGCACTACAGTGGCG^33^ACTCAGATGCATCCAGCCCG^34^CG^35^CTCCAACTGCTCTGATGGCATGGTAA | 357 |
|  | TTACCATACCATCAAAACAATTAAAA | 58 |  |  |
| **PDE4C** | GGGGATAGTTTATGGAGATTTAGTT | 57 | GGGGACAGCTCATGGAGACCCAGCTTGTCG^1^GCCCG^2^CG^3^CAGGGGTTCCCCG^4^ATCCCCG^5^AAGCACCTGTGGGGGCAGCTACAGCG^6^CCCCATCCG^7^CATCCAGCG^8^GCG^9^CTTCCACTCG^10^GACCCAGAGCG^11^CCCTGATCG^12^GCCCG^13^CG^14^CCG^15^GTAAAGTCG^16^CG^17^GCTCTCG^18^TGGCCCAGTTCCTTTCACCG^19^CCG^20^GTAGGTGACG^21^GGGCG^22^GGGCCTATTTGAGGAAATGACCTGTATTGAGAGGGGACACTT | 242 |
|  | AAATATCCCCTCTCAATACAAATCA | 59 |  |  |

**Table C.3 – Mean DNA methylation and standard deviation across the ELOVL2 amplicon in each tissue**

| **ELOVL2 DNA methylation mean** | | | | |
| --- | --- | --- | --- | --- |
| **Tissue** | **Blood % (SD)** | **Lung % (SD)** | **Cerebellum % (SD)** | **Hippocampus % (SD)** |
| **Amplicon average** | 27.72 (3.27) | 16.21 (3.19) | 15.85 (2.80) | 13.8 (2.41) |
| **CpG 1** | 19.64 (3.83) | 8.51 (4.43) | 5.15 (2.13) | 5.42 (3.92) |
| **CpG 2/3** | 30.68 (7.34) | 19.7 (9.51) | 7.47 (10.87) | 9.14 (6.70) |
| **CpG 4** | 11.52 (3.37) | 6.90 (2.52) | 9.67 (5.42) | 8.91 (4.46) |
| **CpG 5** | 29.72 (5.30) | 10.22 (6.08) | 6.31 (11.91) | 3.27 (4.49) |
| **CpG 6** | 28.68 (5.93) | 9.28 (6.91) | 4.31 (3.88) | 3.49 (5.44) |
| **CpG 7/8/9** | 28.50 (5.01) | 14.75 (7.39) | 19.76 (3.32) | 16.20 (6.15) |
| **CpG 10** | 5.24 (2.70) | 3.22 (2.47) | 4.43 (3.24) | 3.43 (4.60) |
| **CpG 11** | 68.52 (6.12) | 57.02 (12.94) | 69.44 (11.74) | 60.40 (13.01) |

**Table C.4 – Mean DNA methylation and standard deviation across the GLRA1 amplicon in each tissue**

| **GLRA1 DNA methylation mean** | | | | |
| --- | --- | --- | --- | --- |
| **Tissue** | **Blood % (SD)** | **Lung % (SD)** | **Cerebellum % (SD)** | **Hippocampus % (SD)** |
| **Amplicon average** | 5.97 (1.17) | 4.94 (1.35) | 4.17 (0.79) | 4.37 (1.54) |
| **CpG 1/2** | 3.86 (2.32) | 3.92 (3.46) | 4.32 (2.73) | 3.62 (3.87) |
| **CpG 4** | 2.97 (1.96) | 2.39 (2.35) | 1.69 (1.14) | 1.73 (1.58) |
| **CpG 6** | 2.14 (3.08) | 1.27 (1.37) | 1.98 (1.80) | 1.69 (2.87) |
| **CpG 7/8/9** | 6.49 (2.03) | 4.66 (1.32) | 3.64 (1.30) | 4.36 (1.81) |
| **CpG 10/11** | 11.80 (3.82) | 8.61 (3.05) | 8.35 (2.41) | 8.85 (3.42) |
| **CpG 12** | 4.90 (2.32) | 5.17 (8.7) | 3.07 (5.74) | 3.96 (10.51) |
| **CpG 13/14** | 11.83 (2.42) | 9.52 (3.44) | 7.15 (4.18) | 7.35 (3.02) |
| **CpG 15** | 2.63 (1.22) | 1.95 (1.37) | 1.98 (0.87) | 1.84 (1.24) |
| **CpG 16** | 7.28 (2.01) | 7.58 (2.59) | 6.00 (1.61) | 6.23 (3.34) |
| **CpG 17** | 5.90 (1.65) | 4.44 (1.32) | 3.53 (1.02) | 3.78 (1.89) |

**Table C.5 – Mean DNA methylation and standard deviation across the MYOD1 amplicon in each tissue**

| **MYOD1 DNA methylation mean** | | | | |
| --- | --- | --- | --- | --- |
| **Tissue** | **Blood % (SD)** | **Lung % (SD)** | **Cerebellum % (SD)** | **Hippocampus % (SD)** |
| **Amplicon average** | 7.42 (1.43) | 7.71 (1.77) | 5.49 (1.84) | 5.70 (3.24) |
| **CpG 1** | 6.53 (3.95) | 0.64 (2.25) | 0.38 (1.12) | 1.34 (4.17) |
| **CpG 2** | 8.83 (6.00) | 1.37 (1.41) | 6.11 (5.05) | 1.82 (4.28) |
| **CpG 3** | 9.52 (3.70) | 13.46 (7.73) | 10.31 (6.66) | 11.30 (20.54) |
| **CpG 4/5** | 7.76 (2.91) | 5.85 (4.06) | 3.37 (3.36) | 3.52 (7.27) |
| **CpG 6** | 11.17 (4.10) | 6.61 (7.04) | 5.71 (4.14) | 4.68 (5.72) |
| **CpG 7/8** | 2.50 (5.50) | 2.82 (8.15) | 2.25 (3.80) | 3.73 (7.68) |
| **CpG 9/10/11** | 22.90 (12.49) | 46.41 (12.79) | 16.55 (13.98) | 20.14 (15.80) |
| **CpG 12** | 1.12 (1.37) | 0.75 (0.84) | 0.35 (0.79) | 0.87 (2.11) |
| **CpG 14** | 11.17 (4.10) | 6.61 (7.04) | 5.71 (4.14) | 4.68 (5.72) |
| **CpG 16** | 10.04 (5.11) | 5.18 (3.63) | 9.21 (5.15) | 4.77 (5.20) |
| **CpG 17** | 1.40 (1.86) | 0.00 (0.00) | 1.57 (3.23) | 0.83 (2.74) |
| **CpG 18** | 5.42 (4.06) | 11.82 (2.58) | 4.50 (3.55) | 10.14 (6.07) |
| **CpG 19** | 10.04 (5.11) | 5.18 (3.63) | 9.21 (5.15) | 4.77 (5.20) |
| **CpG 20** | 9.68 (3.76) | 7.36 (3.06) | 5.93 (4.47) | 6.03 (6.03) |
| **CpG 22/23** | 4.68 (2.57) | 6.76 (5.45) | 3.64 (1.55) | 4.76 (5.55) |
| **CpG 30** | 5.15 (2.90) | 1.85 (1.90) | 5.74 (3.67) | 3.97 (3.64) |
| **CpG 31** | 8.81 (4.43) | 14.87 (6.30) | 4.79 (12.27) | 5.59 (7.43) |
| **CpG 33** | 0.20 (0.55) | 0.35 (1.05) | 0.38 (1.36) | 1.68 (5.32) |
| **CpG 34/35** | 6.27 (2.81) | 7.46 (7.33) | 7.76 (2.88) | 8.73 (6.04) |

**Table C.6 – Mean DNA methylation and standard deviation across the PDE4C amplicon in each tissue**

| **PDE4C DNA methylation mean** | | | | |
| --- | --- | --- | --- | --- |
| **Tissue** | **Blood % (SD)** | **Lung % (SD)** | **Cerebellum % (SD)** | **Hippocampus % (SD)** |
| **Amplicon average** | 5.25 (1.67) | 6.30 (2.15) | 8.58 (2.52) | 8.47 (1.88) |
| **CpG 4** | 13.64 (9.44) | 15.30 (9.33) | 11.01 (6.78) | 20.71 (7.37) |
| **CpG 5** | 5.62 (4.70) | 8.10 (6.17) | 4.79 (2.60) | 8.71 (4.43) |
| **CpG 6** | 2.97 (2.33) | 4.47 (2.32) | 8.62 (10.92) | 4.62 (1.57) |
| **CpG 7** | 4.12 (3.73) | 4.20 (2.95) | 7.18 (4.03) | 4.06 (2.24) |
| **CpG 8/9** | 2.08 (0.90) | 2.42 (1.44) | 4.57 (2.12) | 5.51 (1.45) |
| **CpG 10** | 6.76 (8.69) | 8.45 (9.30) | 8.94 (11.82) | 12.55 (12.65) |
| **CpG 11** | 2.55 (1.18) | 3.42 (1.46) | 7.44 (3.24) | 6.32 (1.50) |
| **CpG 12/13/14/15** | 6.52 (2.88) | 5.22 (1.71) | 9.50 (2.45) | 6.35 (1.54) |
| **CpG 16/17/18** | 4.57 (1.79) | 4.07 (1.56) | 5.90 (1.96) | 4.75 (1.48) |
| **CpG 19/20** | 6.64 (3.67) | 8.51 (5.46) | 11.40 (5.28) | 10.54 (2.86) |
| **CpG 21/22** | 1.77 (1.54) | 4.17 (1.86) | 14.91 (6.74) | 7.91 (1.97) |

**Table C.7 – Age-associated DNA methylation changes across individual CpG sites within the ELOVL2 amplicon.** Regression coefficient (DNA methylation change (%) per day) and *P*-value for age-associated DNA methylation is shown for all CpG units and the amplicon average for each tissue. **P* < 0.05.

| **ELOVL2 age association regression coefficient (*P*-value)** | | | | |
| --- | --- | --- | --- | --- |
| **Tissue** | **Blood** | **Lung** | **Cerebellum** | **Hippocampus** |
| **Amplicon average** | 1.22E-02 (0.01*) | 6.73E-03 (0.02*) | 2.86E-03 (0.18) | -9.20E-04 (0.53) |
| **CpG 1** | 1.40E-02 (0.01*) | 4.56E-03 (0.27) | 1.23E-03 (0.46) | 3.82E-03 (0.10) |
| **CpG 2/3** | 3.88E-02 (1.15E-04*) | 3.70E-03 (0.68) | -7.91E-03 (0.34) | 4.52E-03 (0.28) |
| **CpG 4** | -9.94E-04 (0.85) | 3.64E-03 (0.11) | 9.31E-04 (0.82) | -8.12E-04 (0.77) |
| **CpG 5** | 1.84E-02 (0.02*) | 1.50E-02 (0.01*) | 1.19E-02 (0.19) | -2.28E-03 (0.41) |
| **CpG 6** | 1.20E-02 (0.20) | 1.81E-02 (4.28E-03*) | 8.14E-03 (0.01*) | 5.26E-03 (0.11) |
| **CpG 7/8/9** | 1.08E-02 (0.19) | 1.49E-02 (0.03*) | 3.60E-03 (0.15) | -1.69E-03 (0.66) |
| **CpG 10** | -3.99E-03 (0.35) | 3.25E-03 (0.16) | -4.95E-04 (0.85) | -8.87E-04 (0.76) |
| **CpG 11** | 8.36E-03 (0.36) | -1.22E-02 (0.29) | 4.13E-03 (0.65) | -1.66E-02 (0.03*) |

**Table C.8 - Age-associated DNA methylation changes across individual CpG sites within the GLRA1 amplicon.** Regression coefficient (DNA methylation change (%) per day) and *P*-value for age-associated DNA methylation is shown for all CpG units and the amplicon average for each tissue. **P* < 0.05.

| **GLRA1 age association regression coefficient (*P*-value)** | | | | |
| --- | --- | --- | --- | --- |
| **Tissue** | **Blood** | **Lung** | **Cerebellum** | **Hippocampus** |
| **Amplicon average** | 3.42E-03 (3.86E-05*) | 2.44E-03 (0.01*) | -8.41E-04 (0.16) | 6.53E-04 (0.59) |
| **CpG 1/2** | 5.10E-04 (0.77) | 7.43E-04 (0.78) | -2.14E-03 (0.31) | -3.75E-04 (0.90) |
| **CpG 4** | -4.83E-04 (0.74) | -1.52E-03 (0.39) | 1.34E-03 (0.12) | 1.09E-03 (0.38) |
| **CpG 6** | -1.03E-03 (0.67) | -2.91E-04 (0.78) | -1.68E-03 (0.22) | -2.23E-03 (0.32) |
| **CpG 7/8/9** | 4.67E-03 (2.08E-03*) | 2.64E-03 (0.01*) | -6.66E-04 (0.50) | 2.76E-04 (0.85) |
| **CpG 10/11** | 8.65E-03 (1.96E-03*) | 7.49E-03 (6.30E-04*) | -2.01E-03 (0.28) | 3.48E-03 (0.19) |
| **CpG 12** | 6.15E-03 (2.96E-04*) | 3.50E-03 (0.58) | -5.69E-03 (0.19) | -5.46E-04 (0.95) |
| **CpG 13/14** | 8.00E-03 (3.31E-06*) | 5.25E-03 (0.04*) | 5.07E-03 (0.11) | -2.62E-03 (0.27) |
| **CpG 15** | 3.76E-03 (1.63E-05*) | 9.86E-04 (0.33) | -1.16E-03 (0.08) | 2.10E-03 (0.03*) |
| **CpG 16** | -1.23E-03 (0.43) | 1.06E-03 (0.59) | -4.58E-04 (0.72) | 4.07E-03 (0.12) |
| **CpG 17** | 4.69E-03 (7.59E-05*) | 4.10E-03 (7.83E-06*) | -2.45E-04 (0.75) | 1.84E-03 (0.21) |

**Table C.9 - Age-associated DNA methylation changes across individual CpG sites within the MYOD1 amplicon.** Regression coefficient (DNA methylation change (%) per day) and *P*-value for age-associated DNA methylation is shown for all CpG units and the amplicon average for each tissue. **P* < 0.05.

| **MYOD1 age association regression coefficient (*P*-value)** | | | | |
| --- | --- | --- | --- | --- |
| **Tissue** | **Blood** | **Lung** | **Cerebellum** | **Hippocampus** |
| **Amplicon average** | 1.95E-03 (0.09) | 1.07E-03 (0.45) | 3.04E-03 (0.02*) | 7.93E-05 (0.97) |
| **CpG 1** | 1.35E-02 (2.82E-06*) | 2.75E-03 (0.12) | -5.86E-05 (0.94) | 3.74E-03 (0.19) |
| **CpG 2** | 7.54E-03 (0.12) | 2.65E-04 (0.82) | 3.12E-03 (0.40) | 3.65E-03 (0.21) |
| **CpG 3** | -5.39E-04 (0.85) | 2.95E-03 (0.64) | 1.21E-03 (0.80) | 1.52E-03 (0.91) |
| **CpG 4/5** | 2.75E-03 (0.24) | 1.59E-03 (0.64) | 3.98E-03 (0.10) | 5.93E-03 (0.24) |
| **CpG 6** | 6.26E-03 (0.06) | 5.80E-03 (0.30) | 2.55E-03 (0.40) | 2.31E-03 (0.56) |
| **CpG 7/8** | 2.38E-04 (0.96) | -2.86E-03 (0.69) | 3.84E-03 (0.16) | -7.71E-04 (0.88) |
| **CpG 9/10/11** | -2.71E-02 (0.01*) | -2.94E-03 (0.78) | 2.69E-02 (0.01*) | 1.23E-03 (0.91) |
| **CpG 12** | 2.41E-03 (0.03*) | 1.69E-04 (0.82) | -1.00E-03 (0.12) | -2.76E-03 (0.09) |
| **CpG 14** | 6.26E-03 (0.06) | 5.80E-03 (0.30) | 2.55E-03 (0.40) | 2.31E-03 (0.56) |
| **CpG 16** | -1.87E-04 (0.97) | -1.95E-03 (0.50) | 6.55E-03 (0.10) | -2.72E-03 (0.44) |
| **CpG 17** | 3.19E-03 (0.04*) | 0.00E+00 (NaN) | 3.84E-03 (0.10) | 2.88E-03 (0.13) |
| **CpG 18** | 7.67E-03 (0.02*) | 4.76E-04 (0.82) | 1.37E-03 (0.60) | 1.34E-03 (0.75) |
| **CpG 19** | -1.87E-04 (0.97) | -1.95E-03 (0.50) | 6.55E-03 (0.10) | -2.72E-03 (0.44) |
| **CpG 20** | -4.33E-04 (0.89) | 3.55E-04 (0.88) | 5.39E-03 (0.10) | -8.33E-03 (0.04*) |
| **CpG 22/23** | 2.01E-03 (0.35) | -3.59E-03 (0.53) | 8.50E-04 (0.48) | 2.21E-03 (0.58) |
| **CpG 30** | 4.23E-03 (0.09) | 8.72E-04 (0.57) | 2.25E-03 (0.43) | -3.51E-03 (0.17) |
| **CpG 31** | -4.25E-03 (0.25) | 2.37E-03 (0.68) | -1.12E-02 (0.25) | 1.87E-04 (0.97) |
| **CpG 33** | 7.78E-05 (0.86) | 2.42E-04 (0.77) | -1.56E-04 (0.87) | 2.72E-03 (0.46) |
| **CpG 34/35** | 4.51E-03 (0.04*) | -8.98E-04 (0.88) | -3.39E-03 (0.15) | -4.76E-03 (0.25) |

**Table C.10 - Age-associated DNA methylation changes across individual CpG sites within the PDE4C amplicon.** Regression coefficient (DNA methylation change (%) per day) and *P*-value for age-associated DNA methylation is shown for all CpG units and the amplicon average for each tissue. **P* < 0.05.

| **PDE4C age association regression coefficient (*P*-value)** | | | | |
| --- | --- | --- | --- | --- |
| **Tissue** | **Blood** | **Lung** | **Cerebellum** | **Hippocampus** |
| **Amplicon average** | 2.62E-04 (0.83) | -1.98E-04 (0.89) | 7.43E-03 (5.74E-06*) | -6.27E-04 (0.59) |
| **CpG 4** | -1.26E-02 (0.07) | -3.96E-03 (0.52) | 7.61E-03 (0.11) | 1.38E-03 (0.77) |
| **CpG 5** | -1.11E-03 (0.75) | -4.88E-03 (0.22) | -4.80E-03 (0.01*) | -2.20E-03 (0.43) |
| **CpG 6** | -2.00E-03 (0.24) | 1.90E-03 (0.20) | 1.88E-02 (0.01*) | -4.79E-04 (0.63) |
| **CpG 7** | 4.02E-03 (0.13) | -8.35E-04 (0.66) | 2.04E-03 (0.46) | -2.06E-03 (0.15) |
| **CpG 8/9** | -7.70E-04 (0.26) | -1.27E-04 (0.89) | 4.58E-03 (1.46E-03*) | 6.24E-04 (0.57) |
| **CpG 10** | 8.22E-03 (0.21) | 3.61E-03 (0.55) | 9.83E-03 (0.25) | -5.15E-03 (0.54) |
| **CpG 11** | 4.58E-04 (0.60) | -1.15E-03 (0.22) | 5.55E-03 (0.01*) | -1.87E-04 (0.84) |
| **CpG 12/13/14/15** | 3.82E-03 (0.07) | 8.43E-04 (0.44) | 1.58E-03 (0.36) | -1.27E-04 (0.90) |
| **CpG 16/17/18** | 2.96E-03 (0.02*) | 5.77E-04 (0.57) | 2.80E-03 (0.05*) | 4.35E-04 (0.65) |
| **CpG 19/20** | 4.99E-04 (0.85) | 7.79E-04 (0.83) | 1.17E-02 (1.04E-03*) | -4.49E-04 (0.81) |
| **CpG 21/22** | 1.14E-03 (0.32) | 1.98E-03 (0.10) | 2.07E-02 (2.14E-06*) | 7.89E-04 (0.53) |
